# The New DBpedia Release Cycle: Increasing Agility and Efficiency in Knowledge Extraction Workflows

**DOI:** 10.1007/978-3-030-59833-4_1

**Published:** 2020-10-27

**Authors:** Marvin Hofer, Sebastian Hellmann, Milan Dojchinovski, Johannes Frey

**Affiliations:** 8 Linköping University, Linköping, Sweden; 9 University of Amsterdam, Amsterdam, Noord-Holland The Netherlands; 10 Department of Computer Science, Vrije Universiteit Amsterdam, Amsterdam, Noord-Holland The Netherlands; 11 St. Pölten University of Applied Sciences, St. Pölten, Austria; 12 FIZ Karlsruhe – Leibniz Institute for, Karlsruhe, Germany; 13 Karlsruhe Institute of Technology, Karlsruhe, Germany; 14 UAS St. Pölten, St. Pölten, Niederösterreich Austria; 15 Vienna University of Economics and Business, Vienna, Wien Austria; 16 VU Amsterdam, Amsterdam, The Netherlands; 17 ADAPT Centre, Trinity College Dublin, Dublin, Ireland; 18 Knowledge Integration and Language Technologies (KILT/AKSW), DBpedia Association/InfAI, Leipzig University, Leipzig, Germany; 19 Web Intelligence Research Group, FIT, Czech Technical University in Prague, Prague, Czech Republic

**Keywords:** DBpedia, Knowledge extraction, Data publishing, Quality assurance

## Abstract

Since its inception in 2007, DBpedia has been constantly releasing open data in RDF, extracted from various Wikimedia projects using a complex software system called the DBpedia Information Extraction Framework (DIEF). For the past 12 years, the software received a plethora of extensions by the community, which positively affected the size and data quality. Due to the increase in size and complexity, the release process was facing huge delays (from 12 to 17 months cycle), thus impacting the agility of the development. In this paper, we describe the new DBpedia release cycle including our innovative release workflow, which allows development teams (in particular those who publish large, open data) to implement agile, cost-efficient processes and scale up productivity. The DBpedia release workflow has been re-engineered, its new primary focus is on *productivity* and *agility*, to address the challenges of size and complexity. At the same time, *quality* is assured by implementing a comprehensive testing methodology. We run an experimental evaluation and argue that the implemented measures increase agility and allow for cost-effective quality-control and debugging and thus achieve a higher level of maintainability. As a result, DBpedia now publishes regular (i.e. monthly) releases with over 21 billion triples with minimal publishing effort .

## Introduction

Since its inception in 2007, the DBpedia project [8] has been continuously releasing large, open datasets, extracted from Wikimedia projects such as Wikipedia and Wikidata [15]. The data has been extracted using a complex software system known as the DBpedia Information Extraction Framework (DIEF). Over the past years the system has received a plethora of extensions and fixes by the community which resulted in creating monolithic releases.

Until 2017, The DBpedia release process has been primarily focused on *data quality* and *size*, however, it neglected other two important and desirable goals: *productivity* and *agility* (cf. [3] for balancing the magic triangle on quality, productivity and agility The release process was facing massive delays (from 12 to 17 months) with increasing costs of development and lower productivity due to the sole focus on quality and the increased size and complexity. The releases were so large and complex that the DBpedia core team failed to produce them for almost three years (2017–2019). Note that this was not a performance nor scalability related issue. The DBpedia release workflow has been re-engineered, its new primary focus is on *productivity* and *agility*, to address the challenges of size and complexity. At the same time, the *quality* aspects are assured by implementing a comprehensive testing methodology.

In this paper, we describe the new DBpedia release cycle including our innovative release workflow, which allows development teams (in particular those who publish large, open data) to implement agile, cost-effective processes and scale up productivity. As a result of our innovation DBpedia now produces over 21 billion triples per month with minimal publishing effort.

The paper is organized as follows. First, in Sect. 2, we summarize the two biggest challenges as a motivation for our work, followed by an overview of the release workflow described in Sect. 3. The main process innovations and conceptual design principles are described in Sect. 4. The implemented testing methodology is described in Sect. 5 and the results from several experiments showing the impact, capabilities and the gain from the new release cycle are presented in Sect. 6. Section 7 reports on technologies that relate to ours. Finally, Sect. 8 concludes the paper and presents future work directions.

## Background and Motivation

**1. Agility.** Data quality is one of the largest and oldest topics in computer science independent of current trends such as Big Data or Knowledge Graphs and has a vast amount of facets to consider [16]. Data quality, often defined as *“fitness for use”*, poses many challenges that are frequently neglected or delayed in the software engineering process of applications until the very end, i.e. when the application is demonstrated to the end-user. In this paper, we will refer to this phase of the process as the *“point-of-truth”* since it marks an important transition of **data** (transferred between machines and software) to **information**. At this point, results are presented in a human-readable form so that humans can evaluate them according to their current knowledge and reasoning capacity. We argue that any delay or late manifestation of such a *“point-of-truth”* impacts cost-effectiveness of data quality management and stands in direct contradiction to the first and other principles of the agile manifesto: “Our highest priority is to satisfy the customer through early and continuous delivery of valuable software.” [2]. Our release cycle counteracts the delay by introducing frequent, fixed time-based releases in combination with automated delivery of data to applications via the DBpedia Databus (cf. Subsect. 4.1).

**2. Efficiency.** We focus on efficiency as a major factor of productivity. Data quality follows the Law of Diminishing Returns [11] (similar to Pareto-Efficiency or 80/20 rule), meaning that initially decent quality can be achieved quickly, while complex errors become increasingly much harder to find and fix, up to a point where adding more resources (e.g. human labor or development power) produces similar or worse results^1^. In our experience, there is **no exception to the law of diminishing returns in data**. It affects all data projects, be they collaboratively edited such as Wikidata, semi-automatic such as DBpedia or fully automated machine learning approaches. Additionally, **data quality does usually not depend primarily on the effort invested (e.g. by a large community) but on the efficiency of the development process and the ability to effectively improve data in a sustainable manner**. Measures to increase efficiency are traceability of errors (Subsect. 4.2) combined with testing (Sect. 5).

**Fig. 1.**
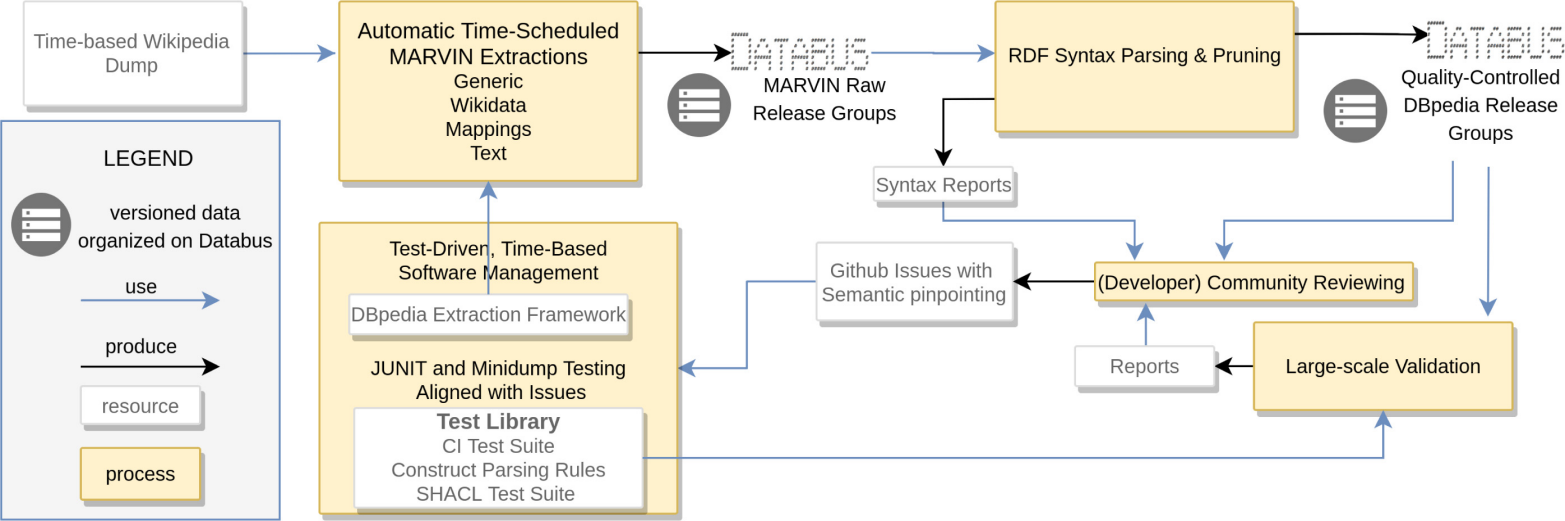
The DBpedia release cycle.

## DBpedia Release Cycle Overview

The DBpedia release cycle is a time-driven release process triggered on a regular basis (i.e. monthly). The DIEF framework (in a distributed computational environment) is executed and data is extracted on the latest Wikipedia dump. The basis of the release cycle relies on the **DBpedia Databus platform**, which acts as a data publishing middleware and is responsible for maintaining information about published data by organizing collection of files as groups and artifacts. The DBpedia Databus is the core component which helps data publishers to publish and promote their data, additionally, it supports data consumers in searching and retrieving data assets. The published file metadata is stored in the Databus repository and is accessible via SPARQL.

**Data Groups and Artifacts.** The process creates five core data groups, each generated by a different extraction process^2^: *i) generic*–information extracted by the generic extractors, *ii) mappings*–information extracted using user specified mapping rules, *iii) text*–extracted Wikipedia article’s content and *iv) ontology*–the DBpedia ontology and *v) wikidata*–extracted and mapped structured data from Wikidata [6]. Each data group consists of one or more versioned data artifacts which represent a particular dataset in different formats, content (e.g. language) and compression variants. In other words, an artifact is a collection of multiple files, which can be addressed with a unique Databus identifier. The artifact IRIs have hierarchical structure and follow a pattern. For example: https://databus.dbpedia.org/dbpedia/mappings/instance-types/2020.04.01.

Where ‘dbpedia’ refers to a publisher, ‘mappings’ refers to a group, ‘instance-types’ refers to an artifact and ‘2020.04.01’ refers to its version.

**Publishing Agents.** A publishing agent acts on behalf of a person or organization and publishes data on the Databus. A Databus account is created and assigned to each agent. The initial set of data groups are published on the Databus by the MARVIN publishing agent.^3^ In addition to the MARVIN agent, there is also the DBpedia agent, which publishes cleaned data artifacts, i.e. syntactically valid. The configuration files used to generate the MARVIN and DBpedia releases are available as a public git repository.^4^

**Cleansing, Validation and Reporting.** The data (i.e., triples) published by the MARVIN agent is then picked up and parsed by the DBpedia agent to create strictly valid RDF triples without any violations (including warnings and errors) based on Apache Jena^5^. Finally, syntactically cleaned data artifacts are published by the DBpedia agent. While the data is syntactically valid, other data quality issues might persist. For example, the IRIs of particular subjects, predicates and objects do not conform to a predefined schema, the data can be structurally incorrect and does not conform to the ontology restrictions, or the release might be incomplete (e.g. missing artifacts). A large-scale validation is done for each release and the error reports are delivered to the community for a review. Figure 1 depicts the overall DBpedia data release cycle.

The complete new DBpedia release approach has been deployed in February 2020. Releases are created every month, except the *text* group, which is released every three months, due to its complexity. We have deployed a light-weight dashboard (see http://release-dashboard.dbpedia.org/) which summarizes the releases, including the extraction process progress, extraction logs and overall statistics. Table 1 provides statistics for different DBpedia data groups for three releases; from Oct 2016,^6^ Aug 2019^7^ and Apr 2020.^8^ ‘2016.10.01’ is the last monolithic legacy release, which we added for comparability. Note that we do not provide numbers for ‘text’ and ‘wikidata’ data groups for the ‘2019.08.30’ due to the incompleteness of these releases.

**Table 1.** Size metrics (i.e. triples count) for DBpedia data groups and release periods.

Version	Generic	Mappings	Text	Wikidata
2016.10.01	4,524,347,267	730,221,071	9,282,719,317	738,329,191
2019.08.30	4,109,424,594	953,187,791	-	-
2020.04.01	3,736,165,682	1,075,837,576	11,200,431,258	4,998,301,802

The numbers from Table 1 show that the amount of triples in the ‘mappings’, ‘text’ and ‘wikidata’ data groups is constantly increasing over time. By contrast, the ‘generic’ data group provides less triples. This is primarily due to the strict testing procedures which have been put in place and as a consequence, invalid statements have been not included in the release. Note that the numbers are also impacted by the configuration of the DIEF system (e.g. enabled extractors) for different releases. Compared to the Wikidata statistic,^9^ the DBpedia ‘wikidata’ extraction produces five times the amount of statements published by itself, mainly because of reification and materialization processes during the extraction (e.g. transitive instance types).

## Conceptual Design Principles

Two design principles have driven the design and implementation of the new DBpedia release cycle: *i) time-driven data releases* enable more frequent and regular DBpedia releases, and *ii) traceability and issue management* enables more efficient linking of issues with tests and tracking their causes.

### Time-Driven vs. Quality-Driven Data Releases

While many of the principles of the agile manifesto are applicable, the most relevant principle “Working software is the primary measure of progress” [2] can not be applied directly to data. As motivated in Sect. 2, the judgment of whether “data works” is withheld until the *“point-of-truth”* on the customer/end-user side. From our own past experience and from conversations with related development teams, it is a fallacy that the developer or data publisher has the capacity to evaluate when “data is useful”, following their own quality-driven or feature-driven agenda. Since adopting an attitude of *“quality creep”*^10^ bears the risk of delaying releases and prevent data reaching end-users with valuable feedback, we decided to switch to a strict time-based schedule for releasing following these principles:

**1. Automated Schedule vs. Self-discipline.** Releases are fully automated via the MARVIN extraction robot. This alleviates developers from the decision when “data is ready”. Else extensive testing of data might have an adverse effect. Developers are prone to “fixing one more bug” instead of delivering data for proper end-user feedback.

**2. Subordination of Software.** The whole software development cycle is completely subordinate to the data release cycle with time-driven, automatic checkout of the tested master branch.

**3. Automated Delivery.** Data is published on the DBpedia Databus, which allows subscription for data (artifacts/versions/files), which in term enables auto-updated application deployment^11^ and therefore facilitating point-of-truth feedback opportunities earlier and continuously.

### Traceability and Issue Management

Any data issues discovered at the point-of-truth start a costly process of backtracking the error in reverse order of the pipeline that delivered it. The problem of tracing and fixing errors becomes even more complicated in Extract-Transform-Load (ETL) procedures where the data is heavily manipulated and/or aggregated from different sources. A quintessential ETL example is the DBpedia system, which implements sophisticated ETL procedures for extraction and refinement of data from semi-structured mixed-quality and crowd-sourced sources such as Wikipedia and Wikidata. Over the years, a huge community of users and contributors has formed around DBpedia, that are reporting errors via different communication channels such as Slack, Github and the DBpedia forum. A vast majority of the issues are associated with i) a piece of data and ii) a procedure (i.e. code) which has generated the data. In the past, the management of issues has been done in an ad-hoc manner. Recently, we introduced a systematic, test-driven approach for managing data and code-related issues using Linked Data. In order to enable more efficient traceability and management of issues, we have introduced two technical improvements:

**1. Explicit Association of Data Artifacts and Code.** Previously DBpedia was grouped by language, which made backtracking difficult. Now every created and published data artifact is explicitly associated, due to a one-time manual mapping, with the procedure (i.e. code) which created the artifact. For example, the “instance-types”^12^ artifact is associated with the “MappingExtractor.scala” class which created the artifact (“View code” action on the Databus website) This allows for easier tracking of errors and relates data to code. A query^13^ on http://databus.dbpedia.org/sparql revealed that 26 code references exist and 12 are still missing for the wikidata group.

**2. Semantic Pinpointing for Issue Management.** A major difficulty for tackling data issues was to identify in which file and version the error occurred. Team-internal discussions as well as submitted community issues did not have the proper vocabulary to describe the datasets, exactly. Using Databus identifiers, these errors can be pinpointed to the exact artifact, version and file.

**Table 2.** Testing methodology levels.

Level	Method	Description
*Software*	JUnit	Functional software tests on data parsers and extractor methods
*Constructs*	Custom rules	IRI patterns and encoding errors, datatype and literal conformity and vocabulary usage
*Syntax*	Syntax parsing	Syntax parsing of output files implemented with Jena with customized selection of applicable errors and warnings
*Shapes*	SHACL	A mix of auto-generated and custom SHACL test suites for domain and value range, cardinality and graph structure
*Integration*	SPARQL over metadata	Verifies completeness of the releases and overall changes of quality metrics using Databus file/package metadata
*Consumer*	SPARQL on graph	Use case and domain specific SPARQL queries at consumer side. Point-of-truth evaluation

**3. Test-Driven Approach for Issue Management (Minidump).** Testing was mostly done after publishing (post-release) and reported issues were often ignored as reproduction of the error were either untraceable or required a full extraction (weeks) and difficult manual intervention. We created a test suite library that can be executed post-release as well as on small-scale, extendable Wikipedia XML dump samples (collection of Wikipedia pages), producing a small release, i.e. a minidump. Tests on this minidump are executed on git push via continuous integration (minutes), thus enabling the following workflow: 1. for each reported data issue, a representative entity is chosen and added to the minidump. 2. a specific test at the appropriate level (see next section) is devised. 3. the code is improved so that the test passes. 4. post-release the same test is executed to check whether the fix was successful at larger scale, also testing for side-effects or breaking other parts of the software.

## Testing Methodology

To cover the entire DBpedia knowledge management life cycle, from software development and debugging to release quality checks, we implemented a robust “Testing Methodology” divided into six different levels listed in Table 2. The first level affects software development only. The following three levels (Constructs, Syntax, and Shapes) are executed on the minidump as well as on the full releases. In comparison, the legacy extraction process did include tests but only covered the testing aspects of the Software and Syntax layers. The continuously updated developer wiki^14^ explains in detail, which steps are necessary to 1. add Construct and SHACL tests, 2. extend the minidumps with entities, 3. configure the Apache Jena-based parser and 4. run the tests and find related code. Besides the improvement in efficiency, the levels of testing were extended to cope with the variety of issues submitted to the DBpedia Issue tracker^15^.

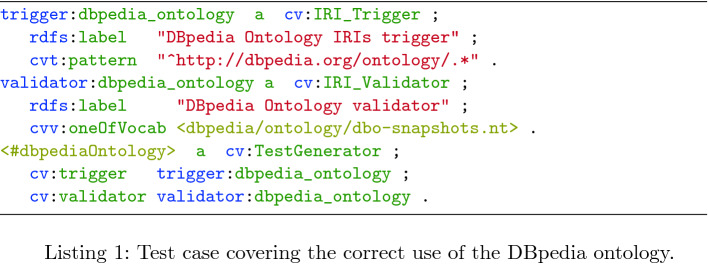



### Construct Validation

To investigate the layout and encoding conformity of produced data, we introduce an approach that focuses on the in-depth validation of its pre-syntactical constructs. This concept differs from *Syntactical Validation*, since it does not rely on the complete syntactical correctness of the analyzed data, but checks the conformity for its single constructs. A construct can be any character or byte sequence inside a data serialization, typically a specific part in the EBNF grammar [12]. In the case of RDF NTriples and DBpedia, interesting constructs are IRIs or literals represented by the subject, predicate, or object part of a single triple. Blank nodes are ignored as they follow unpredictable patterns. Moreover, a single construct can be validated independently of inaccuracies in the rest of the data. This method can be used to gain better test coverage metrics over specific data parts, such as IRI patterns in RDF.

Assessing layout quality of an IRI is motivated by:

1. Linked Data HTTP requests are more lenient towards variation. RDF and SPARQL are strict and require exact match. Especially it is relevant that each release uses the exact same IRIs as before, which is normally not handled in syntactical parsing.
2. optional percent-encoding, especially for international chars and gen/sub-delims^16^ = ‘!’, ‘$’, ‘&’, ‘’’, ‘(’, ‘)’, ‘*’, ‘+’, ‘,’, ‘;’, ‘=’
3. Valid IRIs with wrong namespace **http**://www.wikidata.org/**entity**/Q64 or **https**://www.wikidata.org/**wiki**/Q64 or wrong layout (e.g. wkd:QQ64)
4. Correct use of vocabulary and correct linking

Complementary to *Syntactical Validation*, this approach provides a more fine-grained quality assessment methodology and can be specified as follows:

**Construct Test Trigger:** A *Construct Trigger* describes a pattern (e.g., a regular expression or wildcard) that covers groups of constructs (i.e. namepsaces for IRIs) and assigns them to several domain-specific test cases. Moreover, if a trigger matches a given construct, then it triggers several validation methods that were assigned by a test generator. These patterns are highly flexible, and it is possible to define overlapping triggers.

**Construct Validator:** To verify a group of triggered constructs, a *Construct Validator* describes a specific reusable test approach. Several conformity constraints are currently implemented: *regex* - regular expression matching, *oneOf* - matching a static string, *oneOfVocab* - is contained in the ontology or vocabulary, and *doesNotContain* - does not contain a specific sequence. Further, we implemented generic RDF validators, based on Apache Jena, to test the syntactical correctness of single IRI and literal constructs.

**Construct Test Generator:** A construct test generator defines an 1 : *n* relation between a *Construct Trigger* and several *Construct Validators* to describe a set of test cases.

For our approach, it was convenient to use Apache Spark and line-based regular expressions on NTriples to fetc.h these specific constructs. Listing 1 outlines an example construct test case specification covering DBpedia ontology IRIs, by checking the correct use of defined *owl:Class*, *owl:Datatype*, and *owl:ObjectProperties*. The *Construct Validation* approach seems theoretically extensible to validate namespaces, identifiers, attributes, layouts and encodings in other data formats like XML, CSV, JSON as well. However, we had no proper use case to justify the effort to explore it.

### Syntactical Validation

The procedure of *Syntax Validation* verifies the conformity of a serialized data format with its defined grammar. Normally, RDF parsers distinguish between different levels of"syntactical correctness", including errors and warnings. Errors represent entirely fraudulent statements, in the sense of irreproducible information, and a warning refers to an incorrect format of e.g., a datatype literal.

It is important to validate and clean the produced output of the DIEF, since some of the used methods are bloated, deprecated and erroneous. Therefore, the used *Syntax Validation* is configured to remove all statements containing warnings or errors. This guarantees better interoperability in the target software, which might use parsers considering some warnings as errors. The parser is a wrapper around Apache Jena, highly parallelized and is configured as fault-tolerant to skip erroneous triples and log exceptions correctly. The syntax cleaning process produces strictly valid RDF NTriples, on the one hand, and generates RDF syntax error reports, on the other. The original file is also kept on MARVIN to allow later inspection. The error reports provide structured input for community-driven and automated feedback. Finally, the valid NTriples are sorted to remove duplicated statements. This can later be utilized to compare iterations or modified versions of specific data releases.

### Shape Validation

SHACL (Shapes Constraint Language)^17^ is a W3C Recommendation which defines a language for validating RDF graphs against a set of conditions. These conditions are provided as shapes and other constructs expressed in the form of an RDF graph. SHACL is used within DBpedia’s knowledge extraction and release process to validate and evaluate the results (i.e. generated RDF). The defined SHACL tests are executed against the extracted minidump results (Subsect. 4.2).

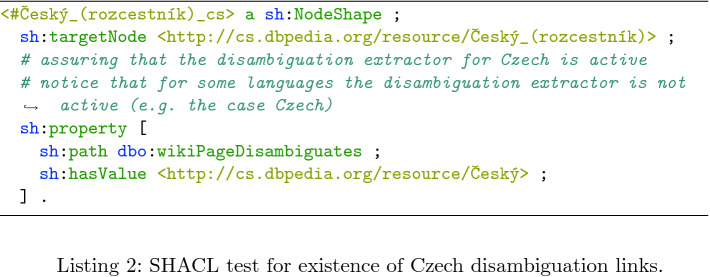



**Motivating Example.** Recently, the Czech DBpedia community identified that the disambiguation links have not been extracted for Czech. The lack was discovered by an application-specific integration test (next section). Upon fixing the problem (configuration-related), a SHACL test (Listing 5.3) was implemented which will in future detect non-existence of the “disambiguation links” dataset on commit by checking a representative triple.

### Integration Validation

Since software and artifacts possess a high coherence and loose coupling, additional methods are necessary to ensure overall quality control. To validate the completeness of a final DBpedia release, we run SPARQL queries on the Databus graph in order to check if all expected files are found. Listing 3 shows an example query to acquire an overview of the completeness of the mappings group releases on the DBpedia Databus.^18^ Other application-specific tests exists, e.g. DBpedia Spotlight needs 3 specific files to compute a language model.^19^

## Experimental Evaluation

Section 3 and Table 1 has already introduced and discussed the size of the new releases. For our experiments, we used the versions listed there and in addition the MARVIN pre-release.

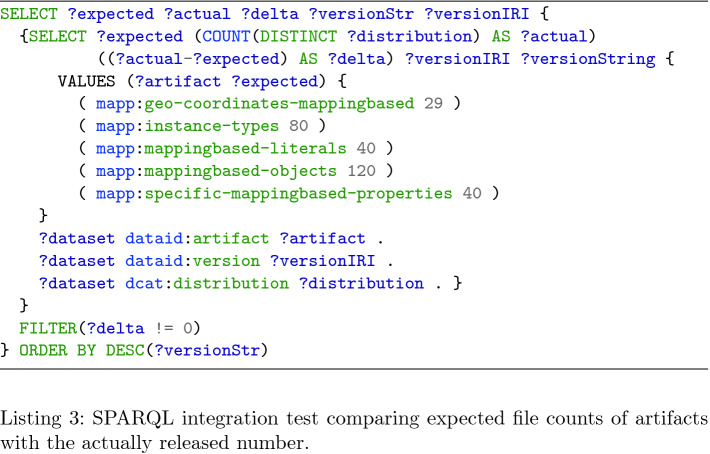



As a variety of methods (e.g. [7], a pre-cursor of SHACL) has been evaluated on DBpedia before and is not repeated here. We focused this evaluation on the novel *Construct Validation*, which introduce a whole previously invisible error class. Results are summarized, detailed reports will be linked to the Databus artifacts in the future. For this paper, they are archived here.^20^

**Construct Validation Tests.** To validate the constructs of the triples produced by DIEF, we specified generic and custom domain-specific test cases. With respect to the constructs in Subsect. 5.1, we provide different test cases for IRI compliance and literal conformity to increase the test coverage over the extracted data. The IRI test cases focus on the encoding or layout of an IRI, and check the correct use of several vocabularies. In case of extracted DBpedia instance IRIs, the test cases validate the correctness considering that a DBpedia resource IRIs should not include sequences of ‘?’, ‘#’, ‘[’, ‘]’, ‘%21’, ‘%24’, ‘%26’, ‘%27’, ‘%28’, ‘%29’, ‘%2A’, ‘%2B’, ‘%2C’, ‘%3B’, ‘%3D’ inside the segment part and follows Wikipedia conventions. The vocabulary test cases, which will be automated later, include tests for these schemas:^21^ dbo, foaf, geo, rdf, rdfs, xsd, itsrdf, and skos to ensure the use of the respective ontology or vocabulary specification. Further, generic IRI and literal test cases are implemented to test their syntactical correctness and to validate the lexical format of typed literals. The full collection of specified custom *Construct Validation* test cases is versioned at the DIEF git repository.^22^

**Construct Validation Metrics**. We define *Construct Validation Metrics* to measure the error rate and the overall test coverage for IRI patterns, encoding errors, datatype formats and vocabularies used in the produced data. The overall construct test coverage is defined by dividing the number of constructs that at least trigger one test by the total amount of found constructs.
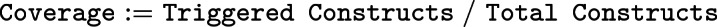


The overall error rate (in percent) is determined by dividing the number of constructs that have at least one error by the total number of covered constructs.
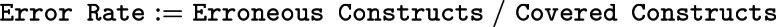

**Table 3.** Custom *Construct Validation* test statistics of the DBpedia and MARVIN release for the generic and mappings group (Gr). Displaying the total IRI counts, the *Construct Validation* test coverage of IRIs, and construct errors (e.g. wrong IRI pattern or vocab usage) of certain Databus releases.

Gr.	Release	Version	IRIs total	Coverage	Errors	Error rate
Generic	DBpedia	2016.10.01	12,228,978,594	83.93%	15,823,204	0.15%
	MARVIN	2019.08.30	11,089,492,791	90.98%	18,113,408	0.18%
	DBpedia	2019.08.30	11,089,492,791	90.98%	18,113,408	0.18%
	MARVIN	2020.04.01	10,527,299,298	89.59%	18,662,921	0.20%
	DBpedia	2020.04.01	10,022,095,645	89.32%	18,652,958	0.21%
Mappings	DBpedia	2016.10.01	2,058,288,765	84.01%	6,692,902	0.39%
	MARVIN	2019.08.30	2,686,427,646	85.99%	6,951,976	0.30%
	DBpedia	2019.08.30	2,678,475,356	86.01%	6,875,930	0.30%
	MARVIN	2020.04.01	3,020,660,756	86.24%	7,514,376	0.29%
	DBpedia	2020.04.01	3,019,942,481	86.24%	7,505,332	0.29%

**Test Results.** The custom tests for the DBpedia ‘generic’ and ‘mappings’ release have an average of 87% IRI coverage (cf. Table 3). The test coverage can be increased by writing more custom test cases, but concerning the 80/20 rule, this could result in high efforts and the missing IRI patterns are presumably used inside of homepage or external link relations. The new strict syntax cleaning was introduced on the ‘2019.08.30’ version of the mappings release and later applied to the ‘generic’ release. It removes a significant amount of IRIs from the ‘generic’ version ($\sim$500 million) and only a fraction from the ‘mappings’ release, reflecting the different extraction quality of them both. Although strict parsing was used and invalid triples are removed, the other errors remain, which we consider a good indicator that the *Construct Validation* is complementary to syntax parsing.

**Table 4.** *Construct Validation* results of the four test cases: XSD date literal (xdt), RDF language string (lang), DBpedia ontology (dbo) and DBpedia Instance URIs which contain a question mark (dbrq). We mention the total number of triggered constructs (prevalence), the aggregated amount of errors, and the percentile error rate.

Gr.	Test	Version	Prevalence	Errors	Error rate
DBp. generic	xdt	2016.10.01	32,104,814	4,419,311	13.77%
		2019.08.30	28,193,951	0	0%
		2020.04.01	26,193,711	0	0%
	lang	2016.10.01	229,009,107	229,009,107	100%
		2019.08.30	353,220,047	353,220,047	100%
		2020.04.01	0	0	0%
DBp. mappings	dbo	2016.10.01	419,745,660	6,686,707	1.594%
		2019.08.30	496,841,363	6,857,202	1.381%
		2020.04.01	567,570,166	7,500,707	1.322%
	dbrq	2016.10.01	853,927,831	0	0%
		2019.08.30	1,198,382,078	15,407	0.001286%
		2020.04.01	1,354,209,107	0	0%

Table 4 shows four independent *Construct Validation* test cases.

**XSD Date Literal (xdt).** This generic triple test validates the correct format use of xsd:date typed literals (  ). Due to the use of strict syntax cleaning, as shown in Table 4, subsequent release later than ‘2016.10.01’ do not contain incorrectly formatted date type literals, loosing several million triples. Removing warnings leads to better interoperability later.

**RDF Language String (lang).** The DIEF uses particular serialization methods to create triples that are often duplicated and contain deprecated code fragments. The post-processing module had an issue to build correct rdf:langString serializations by adding this IRI as explicit datatype instead of the language tag. Considering the N-Triples specification, this is an implicit literal datatype assigned by their language tags. This bug was not recognized by later parsers (i.e. Apache Jena), because the produced statements are syntactically correct. Therefore, to cover this behavior we introduce a generic test case for this kind of literals. The prevalence of this test is described by the pattern ‘  ’ and the test validation is defined by an assertion that the pattern should not exist. Moreover, if a construct can be tested, the test directly fails and so the prevalence of the test is equal to its errors. A post-processing bug fix was provided before the ‘2020.04.01’ release, and considering Table 4 was solved properly.

**DBpedia Ontology URIs (dbo).** To cover correct use of correct vocabularies, some ontology test cases are specified. For the DBpedia ontology this test is assigned to the ‘http://dbpedia.org/ontology/*’ namespace and checks for correctly used IRIs of the DBpedia ontology. The test demonstrates that the used DBpedia ontology instances used inside the three ‘mappings’ release versions do not conform with the DBpedia ontology (cf. Table 4). By inspecting this in detail, we discovered the intensive production of a non-defined class dbo:Location, which is pending to be fixed. Error rate is lower in later releases, as size increased.

**DBpedia Instance URIs (dbrq).** This test case checks one encoding criterion of extracted DBpedia resource IRIs. Therefore, if a construct matches ‘  ’ the last path segment is checked not to contain the ‘?’ symbol as this kind of IRIs should never carry a query part. As displayed in Table 4, the incorrect extraction of the dbr IRIs considering the ‘?’ symbol occurred for version ‘2019.08.30’ and was then solved in later releases.

**Test Coverage of Non-DBpedia Datasets.** To show the re-usability of the *Construct Validation* approach, we analyzed a set of external RDF datasets.^23^ For these datasets our custom test cases achieved an average coverage around 10%. (cf. Table 5). The biggest part is covered by the custom vocabulary tests, especially foaf, rdf, rdfs and skos are commonly used across multiple RDF datasets. Another useful test case represents the correct use of DBpedia IRIs inside these datasets (inbound links). Almost in all external datasets, it could be recognized that backlinked DBpedia instances or ontology IRIs are wrong encoded or incorrectly used. In the case of RDF, this demonstrates that the introduced test approach can validate links between independently produced Linked Open datasets.

**Table 5.** Custom *Construct Validation* statistics and triple counts of external RDF NTriples releases on the Databus. Including IRI test coverage and the number of failed tests based on the custom DBpedia *Construct Validation*.

Dataset	Version	Triples	IRIs Total	Coverage	Errors	Error rate
CaliGraph [4]	2020.03.01	321,508,492	954,813,788	48.50%	30,478,073	6.58%
MusicBrainz [13]	2017.12.01	163,162,562	443,227,356	12.58%	23	0.00%
GeoNames$^\mathrm{a}$	2018.03.11	176,672,577	468,026,653	10.86%	321,865	0.63%
DBkWik [5]	2019.09.02	127,944,902	322,866,512	6.91%	18	0.00%
DNB$^\mathrm{b}$	2019.10.15	226,028,758	502,217,070	3.37%	14	0.00%

$^\mathrm{a}$https://www.geonames.org

$^\mathrm{b}$German National Library - https://www.dnb.de

**Limitations.** *Coverage of Construct Validation.* As demonstrated the *Construct Validation* can test for issues that are not covered by the *Syntax* or *Shape Validation*. But for fine-grained testing, to reach a 100% IRI test coverage on an extracted dataset, it is quite hard to define test cases for every used namespace and vocabulary, concerning their encoding and layouts (e.g., external links). *Comparison of releases.* The number of enabled extractors, produced artifacts, extracted languages, new tests, and mappings can change in newer releases. Therefore, it is challenging to compare evolving releases containing a different set of files and single files that provide more or fewer triples.

## Related Work

At the conceptual level, our work is very related to the “Engineering Agile Big-Data” concepts described in [3] and inspired and based on those particular concepts. Below we discuss the related works to ours and primarily in respect to *i) data release cycle* and *ii) data quality assessment*.

**Data Release Cycle.** The release processes for different knowledge bases are naturally different due to the different ways of obtaining the data. Wikidata, as the most related open data release project, releases dumps on a weekly basis and publishes them in an online file directory^24^ without machine-readable descriptions. In comparison, DBpedia systematically releases data artifacts accompanied with machine-readable descriptions published on the DBpedia Databus platform. This enables data consumers to develop intelligent consumer agents which can easily find and retrieve relevant data artifacts.

Besides Wikimedia, there are other open data release initiatives such as WordNet [9], BabelNet [10] and YAGO [14]. However, all these projects (with exception Wikidata) do not provide regular *time-driven* (e.g. monthly, bi-annual or annual) releases as DBpedia does. Their current release strategy is *feature-driven* and a new data version is released as soon as a new feature or extension has been implemented. This results in delayed and irregular releases. For example, the release of YAGO 4.0 (release in March 2020) took almost three years since the previous YAGO 3.1 release (in June 2017). Similarly, BabelNet^25^ performs feature-driven releases, with the latest BabelNet 4.0 release from Feb 2018 and the previous 3.7 release from Aug 2016.

**Data Quality Assessment.** Further, we briefly mention two projects that attempt Linked Data quality assessment by applying alternative facets.

Due to the different nature, DBpedia implements software/minidump and large-scale validation mechanism. Wikidata performs validation using the Shape Expressions Language (ShEx)^26^ on top of the user generated input.

TripleCheckMate [1] describes a crowd-sourced data quality assessment approach by producing manual error reports of whether a statement conforms to a resource or can be classified as a taxonomy-based vulnerability. Their results showed a broad overview of examined errors but were tied to high efforts and offered no integration concept for further fixing procedures. On the other hand, RDFUnit is a test-driven data-debugging framework that can run automatically generated and manually generated test cases (predecessor of SHACL) against RDF datasets [7]. These automatic test cases mostly concentrate on the schema, whether domain types, range values, or datatypes adhere correctly. The results are also provided in the form of aggregated test reports.

## Conclusion and Future Work

In this paper, we presented and combined several approaches (including *time-based*, *test-driven*, and *traceable* development principles) for increasing the agility and efficiency of knowledge extraction workflows and demonstrated it in the case of the novel DBpedia release cycle. Considering that DBpedia is an enormous open source project, we introduced a new set of extensive test methods, to offer a convenient process for community-driven feedback and development. The DBpedia Databus is used as a quality control interface, due to the utilization of traceable metadata. The *Construct Validation* test approach provides a more in-depth issue tracking checking for wrong formatted datatypes, inconsistent use of vocabularies, and the layout or encoding of IRIs produced in the extracted data. In combination with *Syntactical* and *Shape Validation*, this covers a large spectrum of possible data flaws. Moreover, it was shown that the minidump-based and large-scale test concept provides a flexible view to directly link tests with existing issues. The described workflow builds a reliable and stable base for future DBpedia (or other quality-assured data) releases. However, we presented only a few specific examples of how testing and development of the release process is improved. Therefore, the full potential of how the testing methodologies increase agility and productivity can only be measured after their adoption by the community in the next years. As an overall result, the new DBpedia release cycle produces over 21 billion triples per month with minimal publishing effort. As future work, we will link all created evaluation reports to Databus artifacts, similar to the explained code references (cf. Subsect. 4.2). Further, we plan to extend the usability of the release dashboard.

## Footnotes

1 Meaning overall less output per unit.

2 https://databus.dbpedia.org/dbpedia/.

3 https://databus.dbpedia.org/marvin/.

4 https://git.informatik.uni-leipzig.de/dbpedia-assoc/marvin-config.

5 https://jena.apache.org.

6 https://databus.dbpedia.org/vehnem/collections/dbpedia-2016-10-01.

7 https://databus.dbpedia.org/vehnem/collections/dbpedia-2019-08-30.

8 https://databus.dbpedia.org/vehnem/collections/dbpedia-2020-04-01.

9 https://tools.wmflabs.org/wikidata-todo/stats.php.

10 Analogous to feature creep in software.

11 via Docker, out of scope for this paper, see https://wiki.dbpedia.org/develop/datasets/latest-core-dataset-releases.

12 https://databus.dbpedia.org/dbpedia/mappings/instance-types/2020.04.01.

13 https://git.informatik.uni-leipzig.de/dbpedia-assoc/marvin-config/-/tree/master/paper-supplement/codelink.

14 http://dev.dbpedia.org/Improve_DBpedia.

15 https://github.com/dbpedia/extraction-framework/issues?q=is%3Aissue+is%3Aopen+label%3Aci-tests.

16 https://tools.ietf.org/html/rfc3987.

17 Edited by D. Kontokostas, the former CTO of DBpedia: https://www.w3.org/TR/shacl/.

18 All groups https://git.informatik.uni-leipzig.de/dbpedia-assoc/marvin-config/-/tree/master/test.

19 SPARQL query at https://forum.dbpedia.org/t/tasks-for-volunteers/163 Languages with missing redirects/disambiguations.

20 https://git.informatik.uni-leipzig.de/dbpedia-assoc/marvin-config/-/tree/master/paper-supplement/reports.

21 http://prefix.cc.

22 https://github.com/dbpedia/extraction-framework/blob/master/dump/src/test/resources/dbpedia-specific-ci-tests.ttl.

23 https://databus.dbpedia.org/vehnem/collections/construct-validation-input.

24 https://dumps.wikimedia.org/wikidatawiki/entities/.

25 https://babelnet.org/stats.

26 https://shex.io.
